# Analysis of Inflammation-Related Genes in Patients with Stanford Type A Aortic Dissection

**DOI:** 10.3390/jpm13060990

**Published:** 2023-06-13

**Authors:** Lin Li, Ziwei Zeng, Vugar Yagublu, Nuh Rahbari, Christoph Reißfelder, Michael Keese

**Affiliations:** 1 Department of Vascular Surgery, Medical Faculty Mannheim, Heidelberg University, 68167 Mannheim, Germany; lin.li@medma.uni-heidelberg.de (L.L.); zeng_ziwei123@163.com (Z.Z.); 2 European Center of Angioscience ECAS, Medical Faculty Mannheim, Heidelberg University, 68167 Mannheim, Germany; 3 Surgical Clinic Mannheim, Medical Faculty Mannheim, Heidelberg University, 68167 Mannheim, Germany; vugar.yagublu@umm.de (V.Y.); nuh.rahbari@umm.de (N.R.); christoph.reissfelder@umm.de (C.R.); 4 Department of Vascular Surgery, Theresienkrankenhaus, 68165 Mannheim, Germany

**Keywords:** TAAD, hub gene, inflammation-related genes

## Abstract

**Background:** Aortic dissection (AD) is a life-threatening cardiovascular disease. Pathophysiologically, it has been shown that aortic wall inflammation promotes the occurrence and development of aortic dissection. Thus, the aim of the current research was to determine the inflammation-related biomarkers in AD. **Methods:** In this study, we conducted differentially expressed genes (DEGs) analysis using the GSE153434 dataset containing 10 type A aortic dissection (TAAD) and 10 normal samples downloaded from the Gene Expression Omnibus (GEO) database. The intersection of DEGs and inflammation-related genes was identified as differential expressed inflammation-related genes (DEIRGs). Gene ontology (GO) and Kyoto Encyclopedia of Genes and Genomes (KEGG) pathway analyses were performed for DEIRGs. We then constructed the protein–protein interaction (PPI) network using the Search Tool for the Retrieval of Interacting Genes/Proteins (STRING) database and identified hub genes using the Cytoscape plugin MCODE. Finally, least absolute shrinkage and selection operator (LASSO) logistic regression was used to construct a diagnostic model. **Results:** A total of 1728 DEGs were identified between the TAAD and normal samples. Thereafter, 61 DEIRGs are obtained by taking the intersection of DEGs and inflammation-related genes. The GO indicated that DEIRGs were mainly enriched in response to lipopolysaccharide, in response to molecules of bacterial origin, secretory granule membrane, external side of plasma, receptor ligand activity, and signaling receptor activator activity. KEGG analysis indicated that DEIRGs were mainly enriched in cytokine–cytokine receptor interaction, TNF signaling pathway, and proteoglycans in cancer. We identified *MYC*, *SELL*, *HIF1A*, *EDN1*, *SERPINE1*, *CCL20*, *IL1R1*, *NOD2*, *TLR2*, *CD69*, *PLAUR*, *MMP14*, and *HBEGF* as hub genes using the MCODE plug-in. The ROC indicated these genes had a good diagnostic performance for TAAD. **Conclusion:** In conclusion, our study identified 13 hub genes in the TAAD. This study will be of significance for the future development of a preventive therapy of TAAD.

## 1. Introduction

Aortic dissection (AD), which is defined by the progressive separation of the medial layer and intimal layer of the aorta by the blood entering from the intimal tear, is relatively rare [1]. The annual incidence rate of acute AD is roughly 4.8/100,000 [2]. Depending on the location of the proximal tear, AD is classified as Stanford type A aortic dissection (TAAD) with entry tears in the ascending aorta and Stanford type B aortic dissection (TBAD) with descending aorta entry tears. In total, 40% of patients with TAAD die immediately and mortality is 1–2% for each hour afterwards resulting in a 48 h mortality of approximately 50% in TAAD. Without intervention, up to 90% of patients with acute aortic dissection die within 2 weeks [3]. Rapid diagnosis and decision-making are required when dealing with aortic dissection patients presenting with atypical symptoms such as chest pain, and severe high blood pressure. Thus, there is a need to study novel diagnostic biomarkers and potential therapeutic targets to provide patients with more precise treatment options.

With further research, an increasing amount of evidence identifies the connection between inflammation and both TAAD and TBAD. When AD occurs, inflammatory factors such as interleukin-6 (IL-6) and tumor necrosis factor-α (TNF-α) will be released [4]. In addition to the aortic vascular pathology, these inflammatory factors will cause the patient to develop systemic inflammatory response syndrome (SIRS) or even multiple organ dysfunction syndrome (MODS). Meanwhile, the presence of these inflammatory factors will also affect the structural strength of the aorta. This implies that whether or not interventional treatments or surgery are conducted, the patients will suffer from an increased possibility of complications [4].

Bioinformatic analysis can be used as an effective tool to study gene expression profiles in silico and reveal the underlying molecular mechanism to provide a direction for future research. Therefore, this study aimed to search for inflammation-related markers in TAAD using a combination of bioinformatic methods.

## 2. Materials and Methods

### 2.1. Data Source

GSE153434 and GSE52093 datasets were obtained from the Gene Expression Omnibus (GEO) database. In this study, the GSE153434 dataset was based on the GPL20795 HiSeq X Ten (Homo sapiens) and comprised 10 TAAD and 10 normal samples (Tables S1 and S2) [5]. The GSE52093 dataset was based on the GPL10558 Illumina HumanHT-12 V4.0 expression bead chip and comprised 7 TAAD samples and 5 normal samples (Tables S3 and S4). GSE153434 and GSE52093 were used as training sets and external validation sets, respectively. We obtained 200 inflammation-related genes from the MSigDB database (Table S5) [6].

### 2.2. Screening of DEGs

The DEGs between TAAD and normal samples in the GSE153434 dataset were evaluated using the “DESeq2” [7] R package in the R software (1.4.1023). The parameters |Log2fold change| > 1 and adj. *p* < 0.05 were used as the screening criteria for DEGs. The heatmap and volcano plot were constructed to display DEGs using the “pheatmap” and “ggplot2” packages, respectively. The intersections between DEGs and inflammation-related genes were identified using the “VennDiagram” R package [8] and were defined as DEIRGs for subsequent analysis.

### 2.3. GO and KEGG Analysis

Using the R package “clusterProfiler” (4.8.1) [9], we conducted GO and KEGG analyses for genes. GO terms comprised the biological process (BP), cellular component (CC), and molecular function (MF) [10] and were used to identify the biological properties of genes and gene sets in all organisms. KEGG pathway analysis was performed to obtain the associated enrichment pathways.

### 2.4. Construction of Protein–Protein Interaction (PPI) Network

The STRING database (http://www.string-db.org/, accessed on 1 October 2022) [11] was used to construct a PPI network of the DEIRGs. Cytoscape software (http://www.cytoscape.org, accessed on 1 October 2022) [12] was then used to construct the interaction network map. Subsequently, we used Cytoscape software to screen hub genes using the molecular complex detection (MCODE) plug-in. The semantic similarities of gene classes were calculated using the “GOSemSim” package (2.26.0) [13]. The Corrplot package (0.92) was used to analyze the correlation of hub genes.

### 2.5. Correlation Analysis between Infiltrating Immune Cells and Hub Genes

Immune infiltration analysis was performed by using the GSVA R package (1.48.1) [14]. The ssGSEA was used to quantify the relative infiltration scores of 28 immune cell types. Then, we compared the immune cell infiltration between TAAD and normal samples using Wilcoxon tests. The correlation analysis between hub genes and differential immune cells was calculated via the Spearman method, and the results were visualized. The difference between groups in immune cells was tested using the Wilcoxon and BH (Benjamini and Hochberg) method. BH corrected the *p*-values.

### 2.6. GSEA Analysis

The potential function of the hub genes was analyzed via GSEA using the “clusterprofile” package (4.8.1). The “cp.kegg.v7.1.sh.all.v7.4.symbols.gmt” (2021) reference gene set was downloaded from MSigDB. A nominal *p* < 0.05 was used as the cut-off criteria.

### 2.7. Receiver Operating Characteristic (ROC) Curve Analysis and Expression Analysis

In the GSE153434 dataset, we performed receiver operating characteristic (ROC) curve analysis using the “pROC” R package (1.18.2) [15] on each screened hub genes to verify its accuracy. The hub genes with AUC >  0.7 were deemed useful for disease diagnosis [16]. The hub gene expressions between TAAD and normal in GSE153434 were displayed via a boxplot. The difference between groups in immune cells was tested using the Wilcoxon and BH (Benjamini and Hochberg) method. BH corrected the *p*-values.

### 2.8. Establishment of a LASSO Model

The LASSO diagnostic model was constructed using the “glmnet” R package (4.1-7) based on the gene expression profiles of hub genes. ROC curves, decision curve analysis (DCA), and p-R curves were used to evaluate the effectiveness and accuracy of the model.

## 3. Results

### 3.1. Identification of DEGs/DEIRGs and Functional Enrichment Analysis

**Figure 1:**
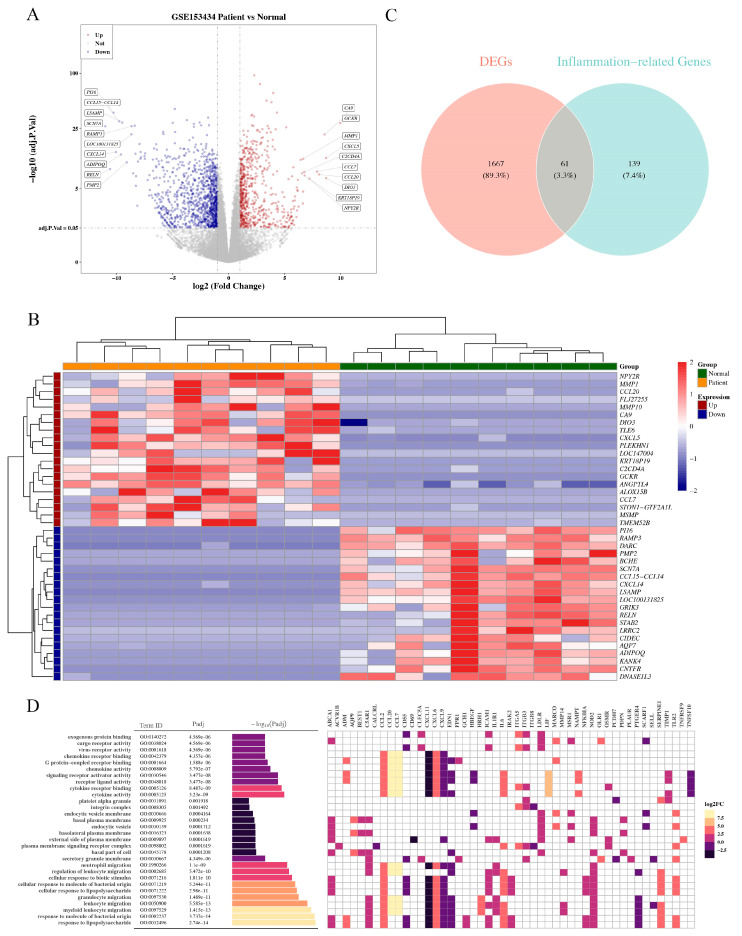
Identification of DEGs/DEIRGs and functional enrichment analysis. (**A**,**B**), Volcano and heatmap of the DEGs. The parameters |Log2fold change| > 1 and adj. *p* < 0.05 were used as the screening criteria for DEGs. Downregulated genes were colored as blue dots in the plots and upregulated genes were colored as red dots. (**C**), Venn diagrams of differential expressed inflammation-related genes (DEIRGs). Red squares indicate positive correlation, whereas blue squares indicate negative correlation. Deeper colors indicate stronger correlation scores. (**D**), The GO terms of the DEIRGs. Heatmap of enriched terms related to the inputted list of genes, colored according to their *p*-values.

**Figure 2:**
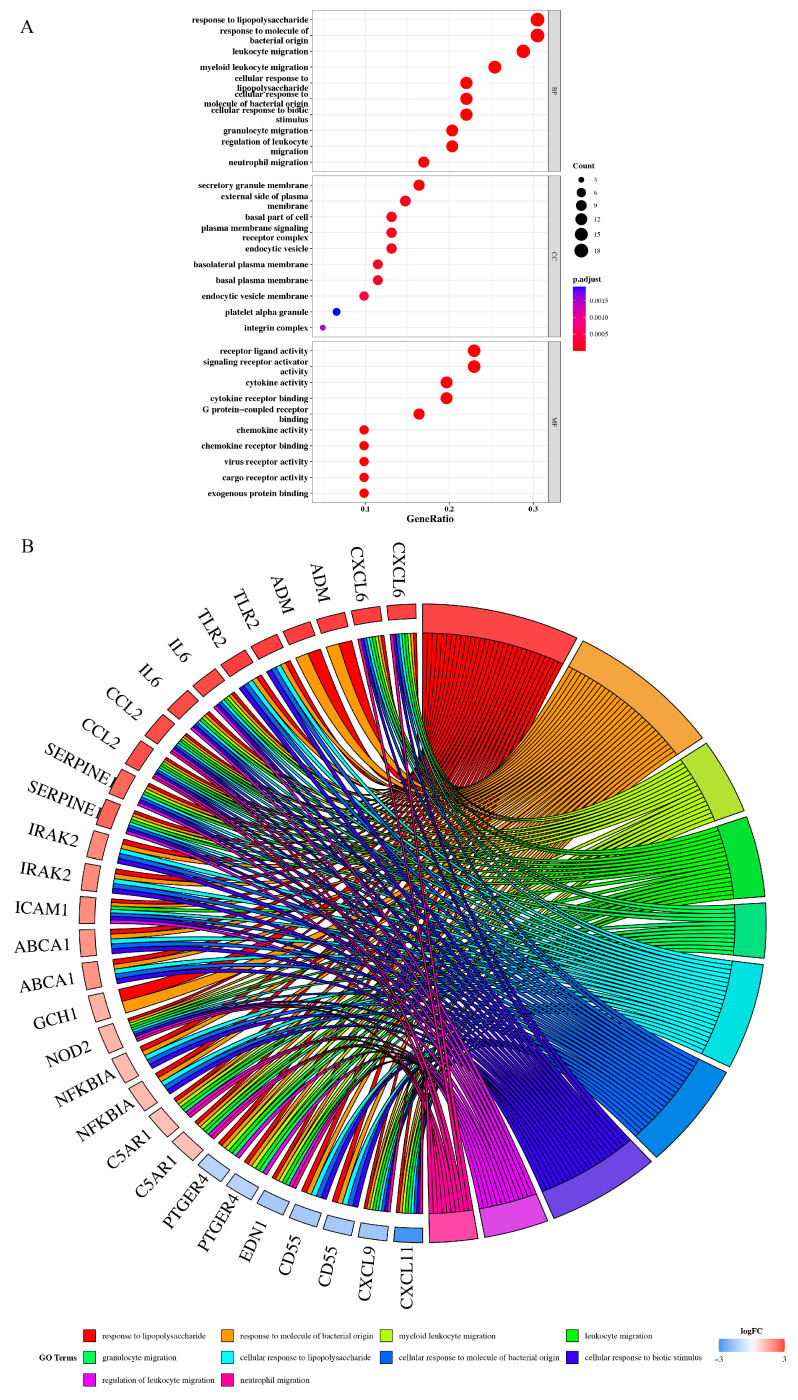
Go terms and enrichment of DEIRGs. (**A**), Advanced bubble chart shows GO enrichment significance items of DEIRGs in three func-tional groups: molecular function (MF), biological processes (BP), and cell composition (CC). (**B**), Chord plot shows the distribution of DEIRGs in different GO-enriched functions. The GO indicated that DEIRGs were mainly enriched in response to lipopolysaccharide, in response to molecules of bacterial origin, secretory granule membrane, and external side of plasma, receptor ligand activity, and signaling receptor activator activity. Gene ratio and pathways are represented by the *x*-axis and *y*-axis, respectively; the size and color of the dots indicate the gene count and the level of *p* value, respectively.

A total of 1728 DEGs were obtained, of which 631 were upregulated and 1097 were downregulated (TAAD vs. normal, Table S6). Volcano and heatmap of the DEGs are shown in Figure 1A,B. Differentially expressed genes exhibit significantly different expression patterns between the TAAD and normal samples. The Venn diagrams showed that 61 genes (DEIRGs) could be obtained (Figure 1C, Table S7). The GO terms of the DEIRGs are shown in Figure 1D. The GO indicated that DEIRGs were mainly enriched in response to lipopolysaccharide, in response to molecules of bacterial origin, secretory granule membrane, and external side of plasma, receptor ligand activity, and signaling receptor activator activity (Figure 2A,B, Table S8).

**Figure 3:**
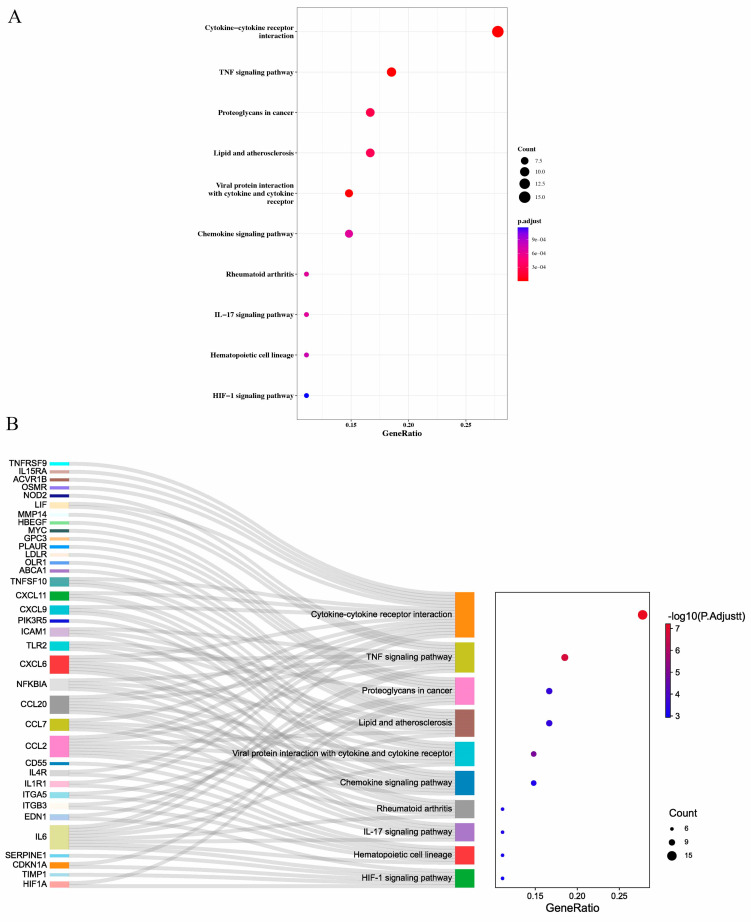
KEGG analysis of DEIRGs enrichments. (**A**), Bubble chart shows KEGG enrichment significance items of DEIRGs. (**B**), Bubble plot com-bined with Sankey diagram demonstrating the enriched KEGG pathways. The KEGG analysis indicated that DEIRGs were mainly enriched in cytokine–cytokine receptor interaction, TNF signaling pathway, and proteoglycans in cancer. Gene ratio and pathways are represented by the *x*-axis and *y*-axis, respectively; the size and color of the dots indicate the gene count and the level of *p* value, respectively.

The KEGG analysis indicated that DEIRGs were mainly enriched in cytokine–cytokine receptor interaction, TNF signaling pathway, and proteoglycans in cancer (Figure 3A,B, Table S9).

### 3.2. Protein–Protein Interaction Network and Hub Gene Identification

**Figure 4:**
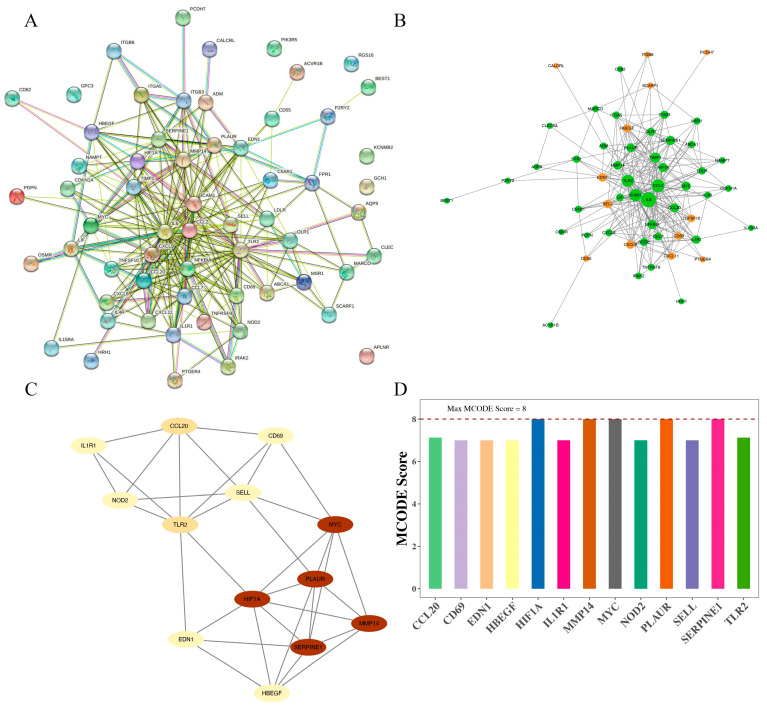
Protein–protein interaction network and hub gene identification. (**A**,**B**), the PPI network of DEIRGs. PPI network of the DEIRGs was constructed with 55 nodes and 262 edges. (**C**), the most highly connected sub-network from the PPI network complex (cluster rank 1; Score 5.667). (**D**), *HIF1A*, *MMP14*, *MYC*, *PLAUR*, and *SERPINE1* were the seed gene with the highest MCODE score in this network.

The PPI network of DEIRGs was established using the STRING database (Figure 4A). The PPI network of the DEIRGs was constructed with 55 nodes and 262 edges (Figure 4B, Table S10). The most highly connected sub-network (cluster rank 1; Score 5.667) was obtained from the PPI network complex (Figure 4C, Table S11), consisting of 13 nodes (*MYC*, *SELL*, *HIF1A*, *EDN1*, *SERPINE1*, *CCL20*, *IL1R1*, *NOD2*, *TLR2*, *CD69*, *PLAUR*, *MMP14*, and *HBEGF*) and 34 interactions, which was therefore considered a critical functional module. *HIF1A*, *MMP14*, *MYC*, *PLAUR*, and *SERPINE1* were the seed gene with the highest MCODE score in this network (Figure 4D).

### 3.3. The Correlation Analysis between the Hub Gene and the Functional Similarity Analysis of the Hub Gene

**Figure 5:**
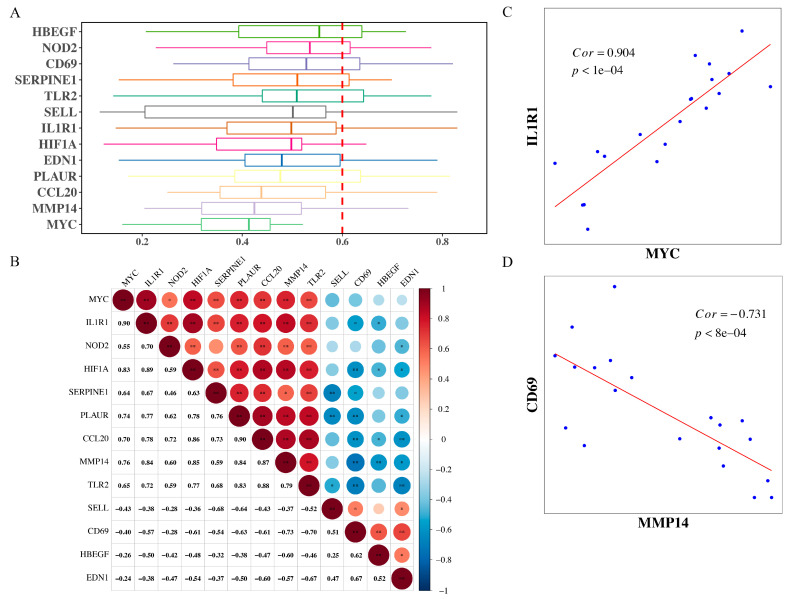
The correlation analysis between the hub gene and the functional similarity analysis of the hub gene. (**A**,**B**), GO semantic similarity of these 13 hub genes. (**C**,**D**), IL1R1 and MYC had the strongest positive correlation (r = 0.904, *p* < 1 × 10^−4^); CD69 and MMP14 had the strongest negative correlation (r = −0.731, *p* < 8 × 10^−4^). Red circles indicate positive correlation, whereas blue circles indicate negative correlation. Deeper colors indicate stronger correlation scores.

We used the “GOSemSim” package in R to calculate the GO semantic similarity of these 13 hub genes in order to further mine for key genes. The higher the semantic similarity, the more important the role that the gene plays in the function. As illustrated in Figure 5A, *CD69*, *NOD2*, and *HBEGF* were the top three genes with the highest-ranking scores. The correlations among those genes were analyzed using GSE153434 (Figure 5B). The correlation results showed that *IL1R1* and *MYC* had the strongest positive correlation (r = 0.904, *p* < 1 × 10^−4^, Figure 5C). *CD69* and *MMP14* had the strongest negative correlation (r = −0.731, *p* < 8 × 10^−4^, Figure 5D).

### 3.4. Correlation Analysis between Hub Genes and Immune Cells

**Figure 6:**
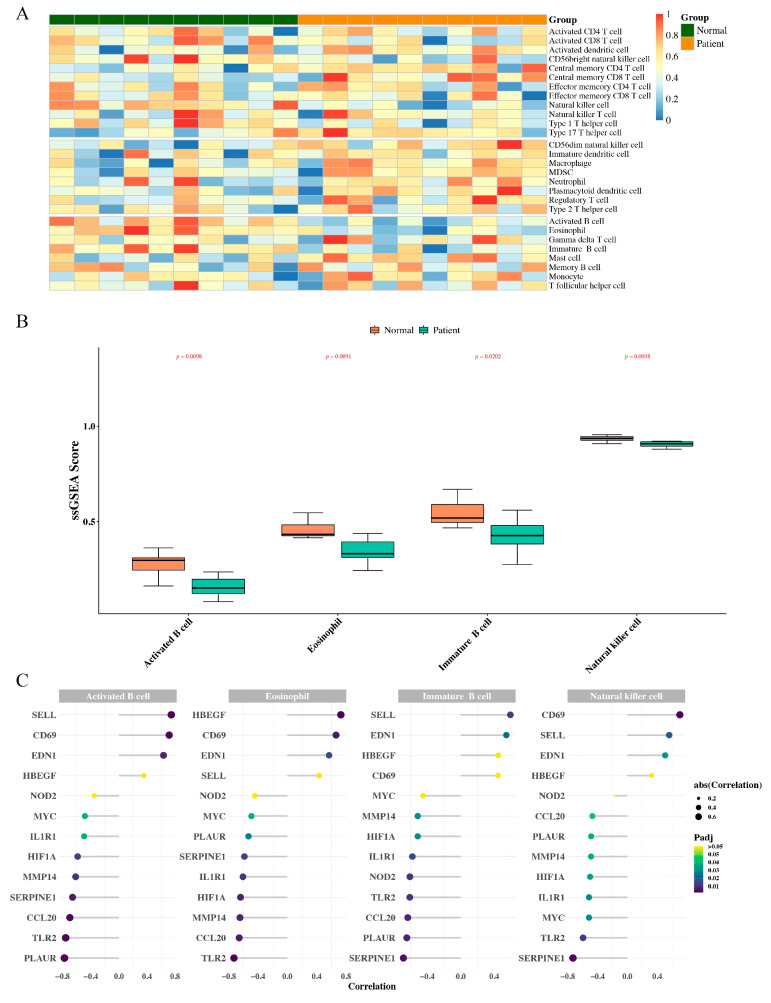
Correlation analysis between hub genes and immune cells. (**A**,**B**), The enrichment fraction of 28 infiltrating immune cells in TAAD and normal sample. The difference between groups in immune cells was tested using the Wilcoxon and BH (Benjamini and Hochberg) method. BH corrected the *p*-values. The results of a total of 4 immune cells were different (adjusted *p*-value < 0.05). (**C**), Correlation analysis between hub genes and differentially immune cells was performed to identify the association of hub genes with immune cell infiltration. The size and color of the dots indicate the Correlation and the level of adjusted *p* value, respectively.

The enrichment fraction of 28 infiltrating immune cells in TAAD and normal samples was presented in the heatmap (Figure 6A). The results in Figure 6B showed that there was a significantly different distribution of activated B cells, eosinophil, immature B cells, and natural killer cells between TAAD and normal samples (*p* < 0.05). Thereafter, correlation analysis between hub genes and differentially immune cells was performed to identify the association of hub genes with immune cell infiltration. The results showed that while the PLAUR was most strongly negatively correlated with activated B cells (r = −0.774, *p* < 0.05), SELL was most strongly positively associated with activated B cells (r = 0.746, *p* < 0.05). While SERPINE1 was most strongly negatively correlated with immature B cells (r = −0.684, *p* < 0.05), SELL was most strongly positively associated with immature B cells (r = 0.592, *p* < 0.05). The TLR2 was most strongly negatively correlated with eosinophil (r = −0.729, *p* < 0.05) and HBEGF was most strongly positively associated with eosinophil (r = 0.729, *p* < 0.05). SERPINE1 also strongly negatively correlated with natural killer cells (r = −0.729, *p* < 0.05). The CD69 was most strongly positively associated with the natural killer cell (r = 0.704, *p* < 0.05) (Figure 6C).

### 3.5. GSEA Analysis

To further determine the potential function of the 13 hub genes, GSEA was performed based on each single hub gene (Supplement Figures S1–S4). By performing GSEA analysis of each hub gene, we found that MYC, HIF1A, EDN1, SERPINE1, CCL20, IL1R1, NOD2, TLR2, PLAUR, MMP14, and HBEGF were related to “KEGG_SPLICEOSOME”, and “KEGG_VASCULAR_SMOOTH_MUSCLE_CONTRACTION”. MYC, HIF1A, EDN1, SERPINE1, CCL20, IL1R1, CD69, PLAUR, MMP14, and HBEGF were associated with “KEGG_PROTEASOME”.

### 3.6. The Expression Analysis and ROC Curve Analysis of Hub Genes

**Figure 7:**
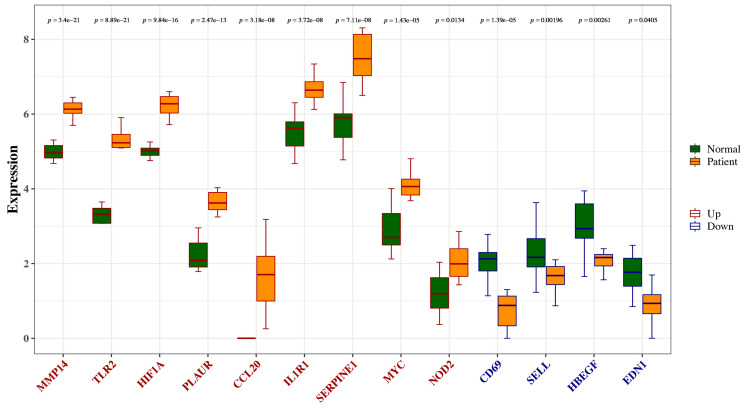
The expression analysis. The difference between groups in immune cells was tested using the Wilcoxon and BH (Benjamini and Hochberg) method. BH corrected the *p*-values.

**Figure 8:**
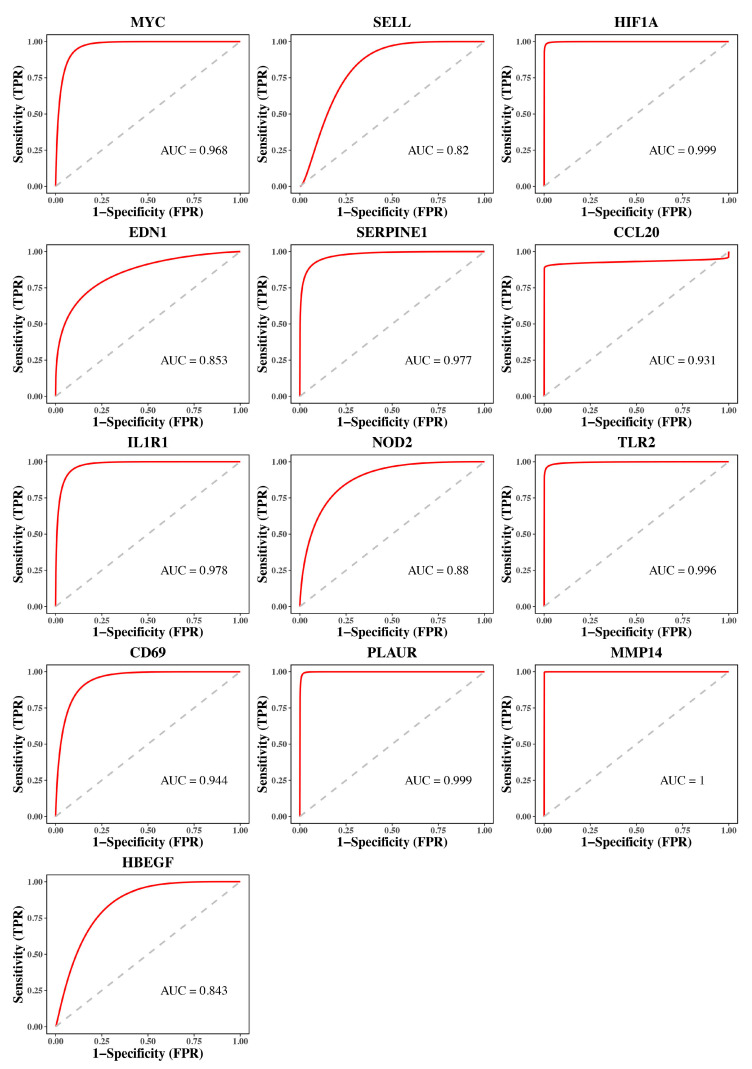
ROC curve analysis of hub genes. The AUC values of 13 genes are all higher than 0.7, demonstrating that these hub genes had good diagnostic values.

We explored the expression of hub genes between TAAD and normal samples and found that HIF1A, MYC, SERPINE1, NOD2, CCL20, IL1R1, TLR2, PLAUR, and MMP14 exhibited higher expression levels in TAAD, while CD69, SELL, HBEGF, and EDN1 exhibited lower expression levels in TAAD (Figure 7). As shown in Figure 8, The AUC values of MYC, SELL, HIF1A, EDN1, SERPINE1, CCL20, IL1R1, NOD2, TLR2, CD69, PLAUR, MMP14, and HBEGF were 0.968, 0.82, 0.999, 0.853, 0.977, 0.931, 0.978, 0.88, 0.996, 0.944, 0.999, 1.000, and 0.843, respectively, demonstrating that these hub genes had good diagnostic values.

### 3.7. LASSO Diagnostic Model for TAAD Was Established Based on Hub Genes

**Figure 9:**
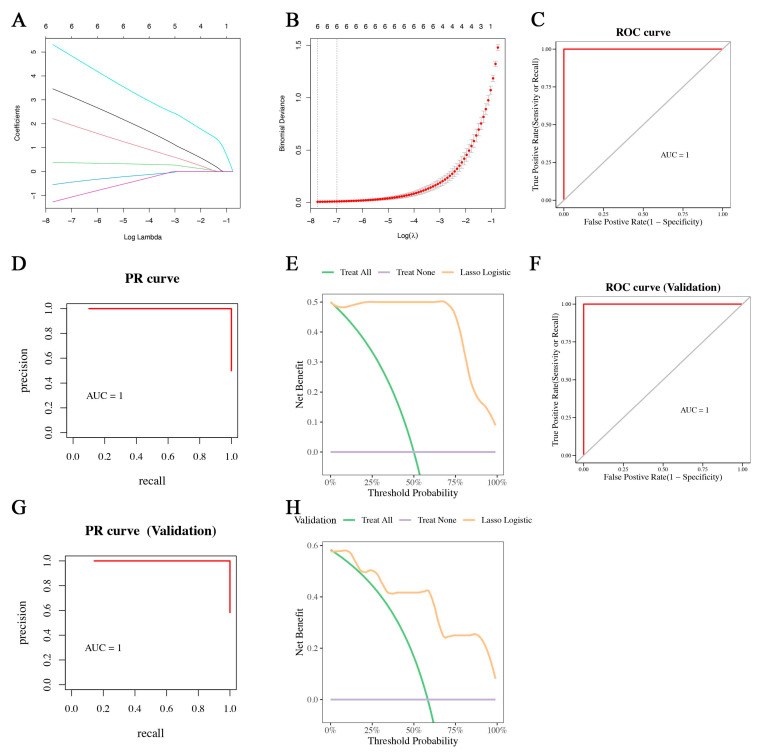
LASSO diagnostic model. (**A**,**B**), Lasso diagnostic model based on HIF1A, SERPINE1, TLR2, CD69, MMP14, and HBEGF. (**C**), ROC curve analysis. (**D**,**E**), PR curve and DCA curve. (**F**–**H**), LASSO diagnostic model was tested in an external dataset.

Thereafter, the LASSO algorithm was used to further screen the gene signature (HIF1A, SERPINE1, TLR2, CD69, MMP14, and HBEGF) of TAAD from hub genes. Thus, we constructed a Lasso diagnostic model based on HIF1A, SERPINE1, TLR2, CD69, MMP14, and HBEGF (Figure 9A,B). The ROC curve indicated that the model had a high accuracy (AUC = 1, Figure 9C). The PR curve and the DCA curves also indicated that this model had good performance (Figure 9D,E). Furthermore, we tested the LASSO diagnostic model in an external dataset (GSE52093). The ROC curve (AUC = 1), the PR curve, and the DCA curve verified that the model maintained high diagnostic accuracy (Figure 9F–H).

## 4. Discussion

With the advancement of aortic dissection studies, the understanding of aortic dissection has evolved from a non-inflammatory to an inflammatory condition. Abundant inflammatory cells can be observed in medial degenerated tissue, such as lymphocytes and macrophages [4]. With the progression of TAAD, damaged aortic wall cells will release a large number of molecules that target and activate the inflammatory cell [17,18]. These cells secrete enzymes such as matrix metalloproteinases (MMPs) that digest extracellular matrix (ECM) and trigger further aortic damage [19].

Our results are derived from the reanalysis of GSE153434. While we have studied inflammation related genes in TAAD in this research, the original study focused on the analysis of 10 autophagy-regulated hub genes that are upregulated in TAAD [5]. Sample collection and processing for RNA extraction had been performed according to standard methods [5]. While the samples were checked macroscopically, no histology has been provided to exclude necrosis of the vessel wall. Using GSE153434 to construct a PPI network, we hereby identified 13 hub inflammation-related genes (*MYC*, *SELL*, *HIF1A*, *EDN1*, *SERPINE1*, *CCL20*, *IL1R1*, *NOD2*, *TLR2*, *CD69*, *PLAUR*, *MMP14*, and *HBEGF*) of TAAD. According to the official description of the STRING database, the interaction score of 0.4 has reached a medium confidence level, while the interaction score of 0.7 will reach a high confidence level. When it is 0.4 or 0.7, or higher, there must be false positives in the results. Therefore, in our study, MCODE was used to identify 13 hub inflammation-related genes. By performing GSEA analysis of each hub gene, we found that the function of hub genes was related to spliceosome, vascular smooth muscle contraction, and the proteasome.

Myelocytomatosis oncogene (*MYC*) and plasminogen activator urokinase receptor (*PLAUR*) were reported to mediate the process of inflammatory disorders with other inflammatory mediators [20,21]. Notably, the expression of MYC is aberrantly activated in the initial stage of pathologic conditions, such as human tumors. Additionally, the upregulation of MYC begins earlier than clinical characteristics [22]. Interleukin-1 receptor 1 (*IL1R1*) coding variant was related to TAAD risk through the accumulation of tumorigenicity 2 receptor (ST2) that may induce inflammatory reactions of the aorta and systemic blood vessels, leading to blood vessel damage in the smooth muscle cells (SMCs) [23]. In our study, *IL1R1* and *MYC* also showed a high diagnostic value and positive correlation with TAAD.

Matrix metalloproteinases (*MMPs*) that cleave interstitial collagens have also been described to play a role in regulating perivascular matrix remodeling in TAAD. A previous study found that the loss of *MMP14* activity increases steady-state vascular leakage [24]. We further found *MMP14* and *CD69* showed the strongest negative correlation in the development of TAAD. Low expression of cluster of differentiation 69 (*CD69*) was found to propagate enhanced inflammatory responses and worsen brain damage after ischemic stroke in animal models through immune cells [25]. Interestingly, here, the expanded natural killer cells showed significant upregulation of *CD69* [26]. Consistently, clinical data from two independent cohorts of patients indicated that increased *CD69* expression in peripheral blood cells after acute myocardial infarction was associated with a lower risk of rehospitalization for heart failure after 2.5 years of follow-up [27]. In concordance, we also found that *CD69* shows a high diagnostic value and interaction with natural killer cells. This might provide a direction for future research on TAAD.

Nucleotide-binding oligomerization domain protein 2 (*NOD2*) was identified as another relevant inflammation-related gene. The expression of *NOD2* can be induced by pro-inflammatory cytokines including TNF-α, IL-1, and IL-6 [28]. Heparin-binding epidermal growth factor (EGF)-like growth factor (*HBEGF*) level was reported as a correlation with the formation of atherosclerosis through regulating *IL-8* expression [29]. Another study found that the deletion of *HBEGF* induced an increase in inflammation and fibrosis in the liver [30]. This indicates the negative correlation between *HBEGF* and inflammation. In our work, we identified a downregulation of *HBEGF*, *EDN1* and *SELL* in the TAAD group. *HBEGF* was of highest diagnostic and therapeutic value. In addition, we found that the function of *HBEGF* was associated with eosinophils, and many factors take part in regulating *HBEGF* expression.

Hypoxia Inducible Factor 1 Subunit: Hypoxia-inducible factors (*HIFs*) are also relevant in TAAD. *HIF1* are activated in response to the hypoxic and inflammatory microenvironment [31]. In the mold of abdominal aortic aneurysm (AAA) mice, macrophage-specific *HIFA* was proven to aggravate the progression of AAA. The study from Lian et al. [32] revealed the role of macrophage *HIF-1α* in vascular inflammation, extracellular matrix degradation, and elastic plate breakage through increased disintegrin and metallopeptidase domain 17 (*ADAM17*). *HIFs* were also reported as mediators of the hypoxic response which regulate more than 2% of genes in vascular endothelial cells either directly or indirectly [33]. Here, *HIF1A* showed high diagnostic and therapeutic value. Even though we have a significant amount of evidence indicating the value of *HIF1A* in vascular diseases, we still lack studies on the role of *HIF1A* in TAAD.

Overexpression of serine protease inhibitor, clade E member 1 (*SERPINE1*), Toll-like receptor 2 (*TLR2*), and CC Chemokine ligand 20 (*CCL20*) were also reported as a significant causative factors in the progression of arteriosclerosis, thrombosis, differentiation of SMCs and perivascular fibrosis [34,35,36]. In our study, we found *TLR2* was most strongly negatively correlated with eosinophils; meanwhile, *SERPINE1* was most strongly negatively correlated with immature B cells and natural killer cells. Our findings are therefore consistent with those of previous studies [37,38,39].

Based on the background mentioned above, in summary, we draw the conclusion that all the 13 hub inflammation-related genes are associated with the inflammation disorders and vascular disorders. It was previously widely acknowledged that the occurrence, progress and prognosis of TAAD were related to an inflammatory response [4]. Thus, as a conclusion to our findings, we assume that the differential expression of 13 hub inflammation-related genes may not only be associated with an inflammatory response but that it may initiate the inflammation of aortic wall, trigger the secretion of inflammatory cytokines and accelerate the dysfunction of SMCs, degeneration of ECM and macrophage polarization. This needs to be validated using in vivo data. If confirmed, these genes may also serve as medication targets in the prevention of TAAD. With the help of the LASSO algorithm, we found *HIF1A*, *SERPINE1*, *TLR2*, *CD69*, *MMP14*, and *HBEGF* were of high diagnostic accuracy. The LASSO diagnostic model based on the selected genes was able to accurately distinguish between TAAD and a normal sample. This illustrates the potential research value of hub genes. Future trials using patient-derived specimens will have to confirm the diagnostic value of *HIF1A*, *SERPINE1*, *TLR2*, *CD69*, *MMP14*, and *HBEGF* for patients with TAAD. In clinical practice, the introduction of index system is helpful for the evaluation of the severity of cardiovascular disorders [40,41]. Additionally, many studies identified the potential biomarkers for AD, such as matrix metalloproteinases, TGF-β, soluble elastin fragments, smooth muscle myosin heavy chain, creatine kinase, calponin, d-dimer, platelets and C-reactive protein. These biomarkers are verified to increase in patients affected with AD [42]. These biomarkers provide an early and fast detection of AD. In recent years, with the help of quantitative proteomics assays, the early detection of proteins involved in inflammation, extracellular matrix remodeling, and cell matrix interactions in serum samples from patients has become possible [43,44]. These biomarkers will highly improve diagnostic accuracy. Further studies will focus on the connection of these biomarkers with the progression of AD. The enrollment of these biomarkers will make the index system more reliable. In future research, *HIF1A*, *SERPINE1*, *TLR2*, *CD69*, *MMP14*, and *HBEGF* can also be used to construct a new inflammation scoring system in combination with other clinical indexes. In conclusion, our study investigated the diagnostic value of inflammation-related genes in TAAD and investigated TAAD-specific regulation of key inflammation-related genes in TAAD. Further experimental investigation of these indicators may yield new diagnostic and therapeutic targets for TAAD patients to demonstrate the significance of this work. As with any in silico study, our work has shortcomings. Firstly, our study is based on public transcriptome sequencing datasets that provide limited information. More sequencing data with in vitro and in vivo experimental verification will be required in our future studies. Secondly, the current analysis is based on transcription level. Conformational experiments that demonstrate a corresponding protein expression are also required. Furthermore, most importantly, we cannot compensate for sample quality problems that produced the bulk transcriptome sequencing datasets.

## Data Availability

All datasets of this study are available in the GEO database (https://www.ncbi.nlm.nih.gov/geo/, GSE153434 and GSE52093, accessed on 1 October 2022).

## Supplementary Materials

The following supporting information can be downloaded at: https://www.mdpi.com/article/10.3390/jpm13060990/s1, Table S1: GSE153434_all genes expression; Table S2: GSE153434_phenotype; Table S3: GSE52093_genes expression; Table S4: GSE52093_phenotype; Table S5: inflammation genes; Table S6: DEGs; Table S7: DEIRGs; Table S8: GO; Table S9: KEGG; Table S10: string_interactions; Table S11: mcode_cluster; Figure S1–S4: GSEA analysis for 13 hub inflammation-related genes.
