# Supplementary figures and images for: Analysis of Inflammation-Related Genes in Patients with Stanford Type A Aortic Dissection
Source: J Pers Med. 2023 Jun 13;13(6):990. doi: 10.3390/jpm13060990 (PMC10302091; doi:10.3390/jpm13060990)

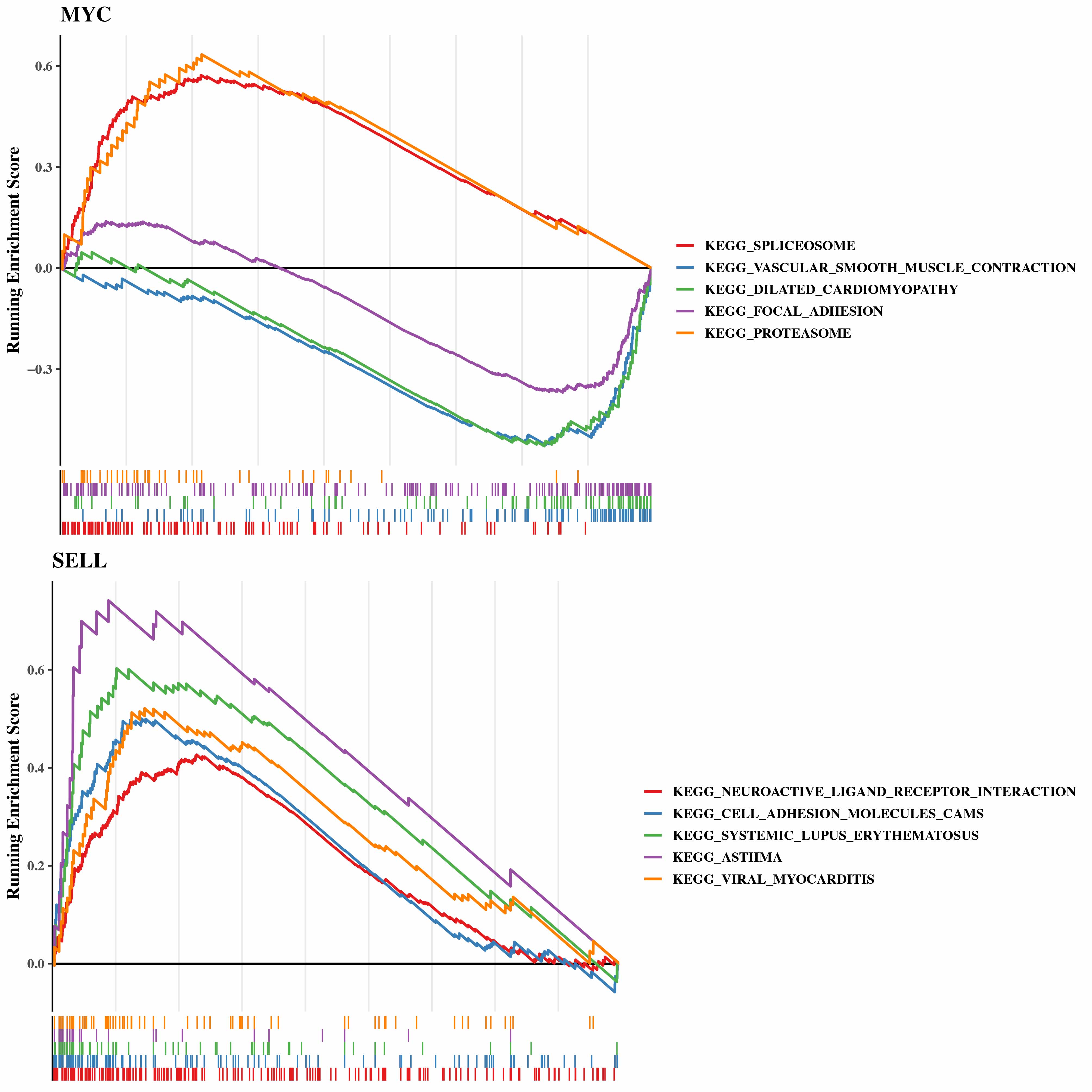

Supplement: Supplementary file 1 [file jpm-13-00990-s001.zip › Figure S1.jpg]

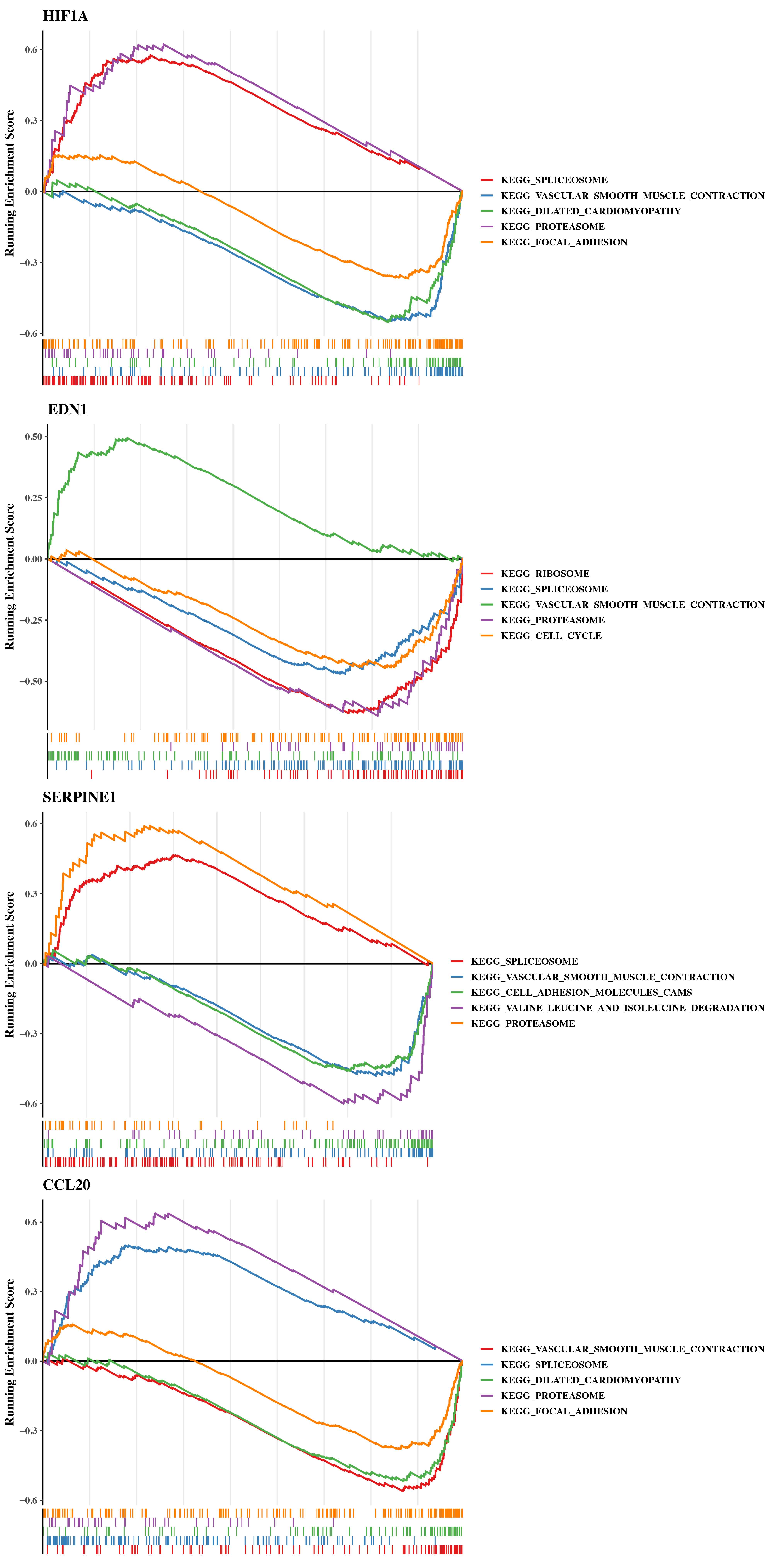

Supplement: Supplementary file 1 [file jpm-13-00990-s001.zip › Figure S2.jpg]

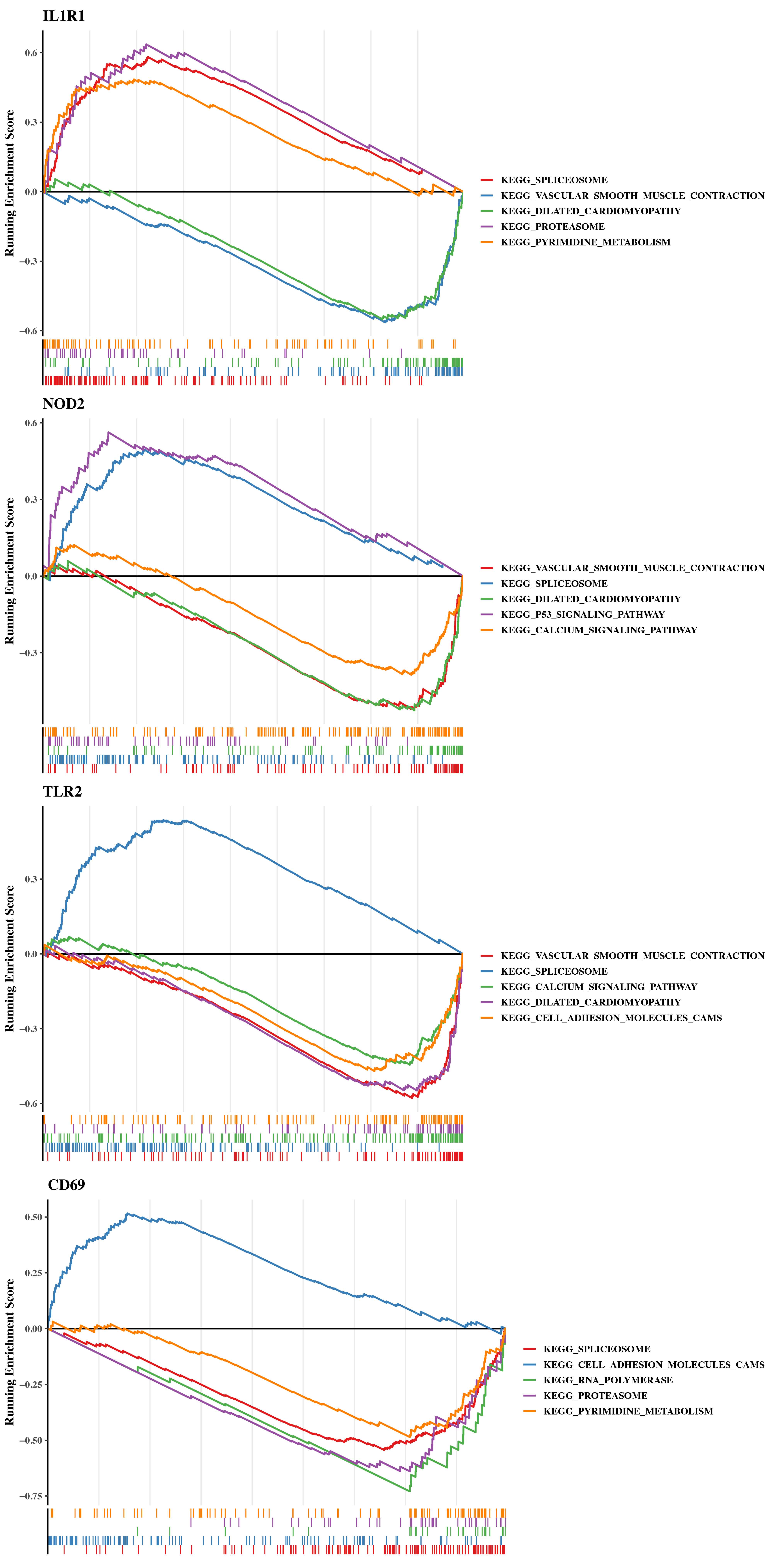

Supplement: Supplementary file 1 [file jpm-13-00990-s001.zip › Figure S3.jpg]

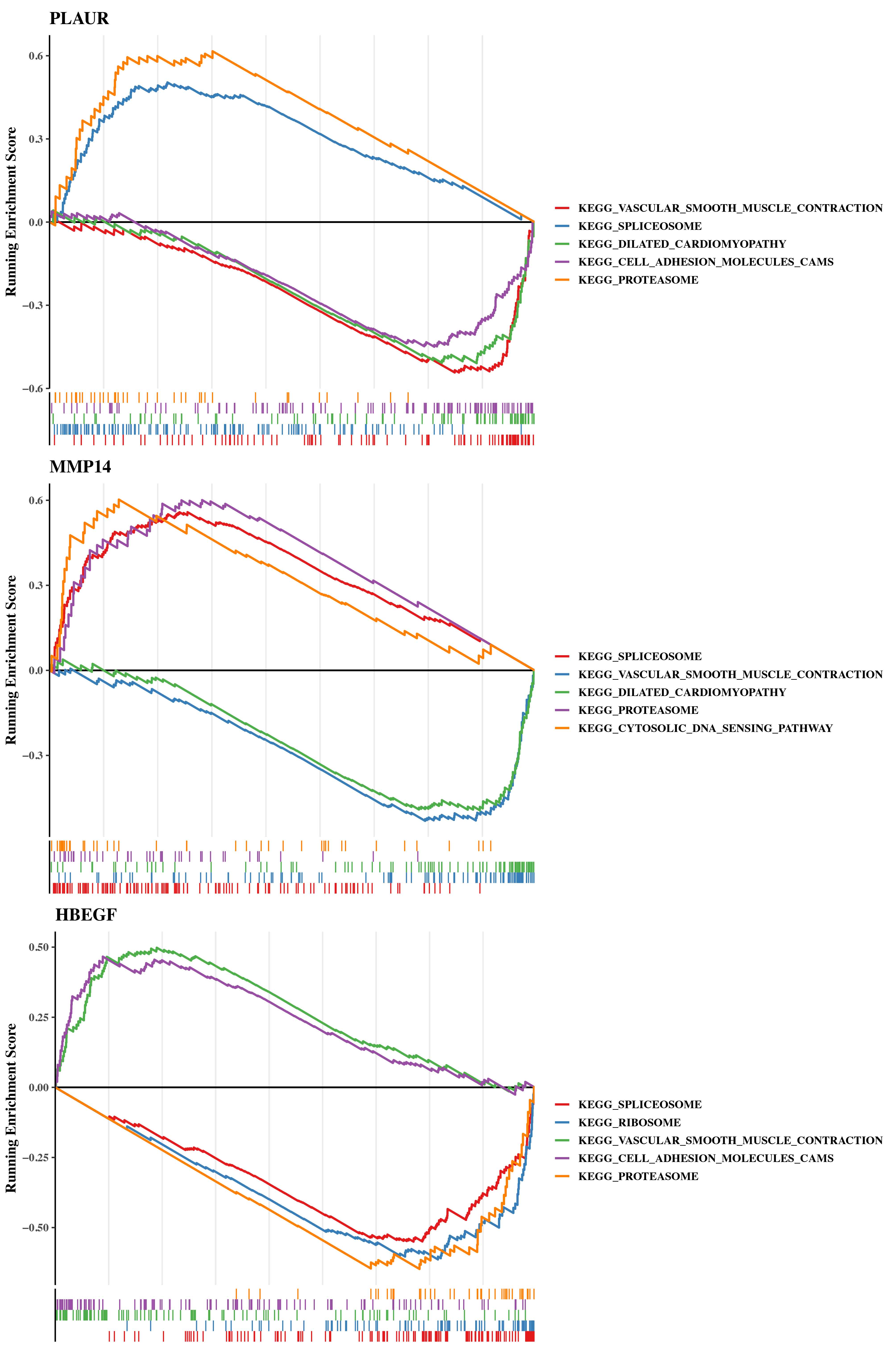

Supplement: Supplementary file 1 [file jpm-13-00990-s001.zip › Figure S4.jpg]
